# Supplementary material for: Higher Accumulation of Visceral Adipose Tissue Is an Independent Risk Factor for Hepatocellular Carcinoma among Viral Hepatitis Patients with Non-Cirrhotic Livers
Source: Cancers (Basel). 2021 Nov 28;13(23):5980. doi: 10.3390/cancers13235980 (PMC8657094; doi:10.3390/cancers13235980)
Supplement: Supplementary file 1 [file cancers-13-05980-s001.zip › cancers-1475412-supplementary.pdf]

Supplementary Materials: Higher Accumulation of Visceral Adipose Tissue is an Independent Risk Factor for Hepatocellular Carcinoma among Viral Hepatitis Patients with Non-Cirrhotic Livers

Kenji Imai, Koji Takai, Takao Miwa, Toshihide Maeda, Tatsunori Hanai, Yohei Shirakami, Atsushi Suetsugu and Masahito Shimizu

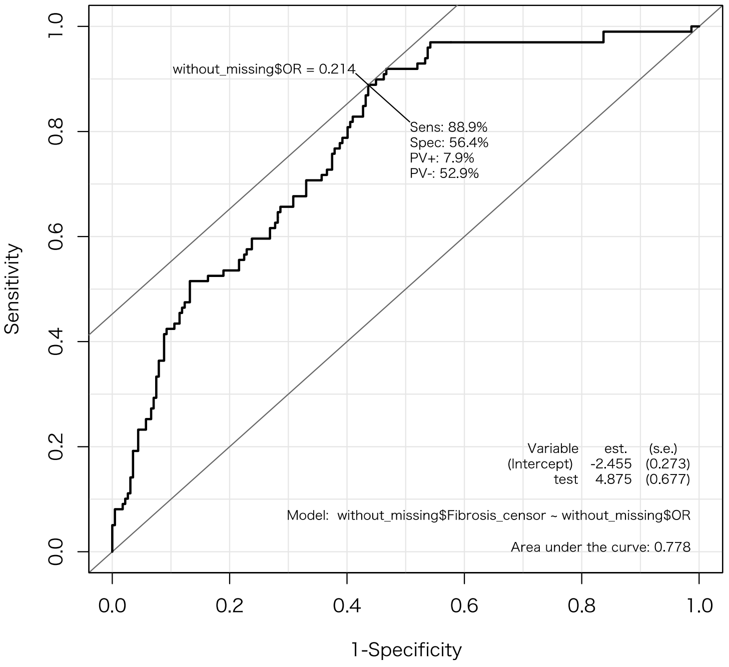

Figure S1. The receiver operating characteristic curve in evaluating the optimal cut-off value of the odds ratio of HCC in a non-cirrhotic liver.

Table S1. The details of the parameters used in the predictive formula for the odds ratio of HCC in a non-cirrhotic liver.

Valuabels	Estimated Parameter	Standardized Error	*p* Value
Intercept	−0.68	1.31	0.603
Age (years)	−0.03	0.02	0.033
Sex (male vs. female)	1.15	0.36	0.001
VATI (cm^2/m^2)	0.01	0.01	0.038
HbAic (%)	0.26	0.13	0.039
HTN (yes vs. no)	0.72	0.29	0.014
Etiology (HBV vs. HCV)	−1.39	0.35	<0.001

HCC, hepatocellular carcinoma; VATI, visceral adipose tissue index; HbA1c, hemoglobin A1c; HTN, hypertension; HBV, hepatitis B virus; HCV, hepatitis C virus

Table S2. Age-adjusted multivariate logistic regression analyses of pathogenic risks of HBV/HCV-related HCC in a non-cirrhotic liver.

Variables	Univariate Analysis		Multivariate Analysis	
	**HR (95% CI)**	***p* value**	**HR (95% CI)**	***p* value**
Sex (male vs. female)	2.74 (1.47–5.10)	0.002	3.15 (1.56–6.36)	0.001
BMI (kg/m^2)	1.01 (0.94–1.08)	0.850		
SMI (cm^2/m^2)	1.01 (0.98–1.04)	0.447		
SATI (cm^2/m^2)	1.00 (0.99–1.01)	0.836		
VATI (cm^2/m^2)	1.02 (1.01–1.03)	<0.001	1.01 (1.00–1.03)	0.038
Cancer stage (I/II/III/IV)	1.14 (0.89–1.46)	0.289		
FPG (mg/dL)	1.00 (0.99–1.01)	0.187		
HOMA-IR	0.96 (0.87–1.06)	0.388		
HbA1c (%)	1.39 (1.11–1.76)	0.006	1.30 (1.01–1.67)	0.040
TG (mg/dL)	1.00 (1.00–1.01)	0.076		
DM (yes vs. no)	1.09 (0.64–1.87)	0.748		
HL (yes vs. no)	2.14 (0.62–7.41)	0.228		
HTN (yes vs. no)	2.42 (1.44–4.07)	<0.001	2.06 (1.16–3.67)	0.014
Etiology (HBV vs. HCV)	3.17 (1.69–5.95)	< 0.001	4.00 (2.02–7.94)	< 0.001

HBV, hepatitis B virus; HCV, hepatitis C virus; HCC, hepatocellular carcinoma; HR, hazard ratio; CI, confidence interval; BMI, body mass index; SMI, skeletal muscle index; SATI, subcutaneous adipose tissue index; VATI, visceral adipose tissue index; FPG, fasting plasma glucose; HOMA-IR, homeostasis model assessment-insulin resistance; HbA1c, hemoglobin A1c; TG, triglyceride; DM, diabetes mellitus; HL, hyperlipidemia; HTN, hypertension.
